# *c*-Type Cytochrome-Dependent Formation of U(IV) Nanoparticles by Shewanella oneidensis

**DOI:** 10.1371/journal.pbio.0040268

**Published:** 2006-08-08

**Authors:** Matthew J Marshall, Alexander S Beliaev, Alice C Dohnalkova, David W Kennedy, Liang Shi, Zheming Wang, Maxim I Boyanov, Barry Lai, Kenneth M Kemner, Jeffrey S McLean, Samantha B Reed, David E Culley, Vanessa L Bailey, Cody J Simonson, Daad A Saffarini, Margaret F Romine, John M Zachara, James K Fredrickson

**Affiliations:** **1**Biological Sciences Division, Pacific Northwest National Laboratory, Richland, Washington, United States of America; **2**Chemical Sciences Division, Pacific Northwest National Laboratory, Richland, Washington, United States of America; **3**Biosciences Division, Argonne National Laboratory, Argonne, Illinois, United States of America; **4**Advanced Photon Source, Argonne National Laboratory, Argonne, Illinois, United States of America; **5**Department of Biological Sciences, University of Wisconsin, Milwaukee, Wisconsin, United States of America; The Institute for Genomic ResearchUnited States of America

## Abstract

Modern approaches for bioremediation of radionuclide contaminated environments are based on the ability of microorganisms to effectively catalyze changes in the oxidation states of metals that in turn influence their solubility. Although microbial metal reduction has been identified as an effective means for immobilizing highly-soluble uranium(VI) complexes in situ, the biomolecular mechanisms of U(VI) reduction are not well understood. Here, we show that *c*-type cytochromes of a dissimilatory metal-reducing bacterium, Shewanella oneidensis MR-1, are essential for the reduction of U(VI) and formation of extracelluar UO _2_ nanoparticles. In particular, the outer membrane (OM) decaheme cytochrome MtrC (metal reduction), previously implicated in Mn(IV) and Fe(III) reduction, directly transferred electrons to U(VI). Additionally, deletions of *mtrC* and/or *omcA* significantly affected the in vivo U(VI) reduction rate relative to wild-type MR-1. Similar to the wild-type, the mutants accumulated UO _2_ nanoparticles extracellularly to high densities in association with an extracellular polymeric substance (EPS). In wild-type cells, this UO _2_-EPS matrix exhibited glycocalyx-like properties and contained multiple elements of the OM, polysaccharide, and heme-containing proteins. Using a novel combination of methods including synchrotron-based X-ray fluorescence microscopy and high-resolution immune-electron microscopy, we demonstrate a close association of the extracellular UO _2_ nanoparticles with MtrC and OmcA (outer membrane cytochrome). This is the first study to our knowledge to directly localize the OM-associated cytochromes with EPS, which contains biogenic UO _2_ nanoparticles. In the environment, such association of UO _2_ nanoparticles with biopolymers may exert a strong influence on subsequent behavior including susceptibility to oxidation by O _2_ or transport in soils and sediments.

## Abstract 2

Microorganisms that catalyze changes in oxidation states of metals can be used to decontaminate environments of radionuclides. Here, *c*-type cytochromes on the outer membrane of a dissimilatory metal reducing bacterium are shown to be essential for the reduction of uranium(VI).

## Introduction

Dissimilatory metal-reducing bacteria (DMRB) constitute a phylogenetically diverse group that spans from hyperthermophilic Archaea to anaerobic Proteobacteria [ 1, 2]. Among those, species of the *Geobacter* and *Shewanella* genera are the most intensively studied metal-reducers, whose hallmark feature is a remarkable respiratory versatility [ 1, 2]. Under anaerobic conditions, these organisms reduce a variety of organic and inorganic substrates, including fumarate, nitrate, nitrite, and thiosulfate as well as various polyvalent metal ions either as soluble complexes or associated with solid phase minerals. These metals include cobalt, vanadium, chromium, uranium, technetium, plutonium, iron, and manganese [ 2– 6].

The ability to utilize such a wide array of electron acceptors is largely due to the diversified respiratory network found in Shewanella oneidensis MR-1, in which the *c*-type cytochromes constitute the integral part of the terminal reductase complexes. Analysis of the genome sequence of S. oneidensis MR-1 indicated that this organism contains 42 putative *c*-type cytochrome genes including many multi–heme-containing proteins [ 7]. In gram-negative bacteria, the terminal reductases, including *c*-type cytochromes, are typically located in the cytoplasmic membrane or the periplasm [ 8]. An unusual feature of organisms like *Shewanella* and *Geobacter* that allows these species to access insoluble metal electron acceptors is the production of high-molecular-weight *c*-type cytochromes reported to be in association with the outer membrane (OM) [ 9– 14]. Cell fractionation of S. oneidensis MR-1 grown under anaerobic conditions demonstrated that approximately 80% of the membrane-bound *c*-type cytochromes were associated with OM cell fractions [ 13]. Subsequent mutagenesis studies in MR-1 have identified a cluster of three metal reduction-specific genes, *mtrC* (locus tag: SO1778), *mtrA* (SO1777), and *mtrB* (SO1776), encoding a putative OM decaheme *c*-type cytochrome, a periplasmic decaheme *c*-type cytochrome, and an OM protein of unknown function, respectively [ 8, 15]. Further analysis of S. oneidensis MR-1 genome has revealed the presence of three other clusters similar to *mtrAB* and three genes homologous to *mtrC* [ 10]. One of these genes encodes a decaheme *c*-type cytochrome, designated OmcA (SO1779), which was subsequently isolated and sequenced [ 16]. Both MtrC and OmcA have been shown to be exposed on the outer face of the OM [ 17], allowing them to contact extracellular soluble and insoluble electron acceptors.

Among many metal and radionuclide contaminants, uranium (U) is one of the primary concerns at U.S. Department of Energy sites because it typically exists as a soluble U(VI) carbonate complex in oxidized, circumneutral pH groundwater. However, U(VI) is readily reduced by DMRB under anoxic conditions resulting in the precipitation of uraninite (UO _2_) [ 18, 19]. The rapid rate of U(VI) reduction by DMRB [ 20] and the relatively low solubility of U(IV) make bioreduction an attractive remedy for removing soluble U(VI) from contaminated groundwater [ 21– 23]. We have previously demonstrated the reduction and extracellular accumulation of UO _2_ precipitates at the OM surface and within the periplasmic space of S. putrefaciens strain CN32 [ 5, 24, 25]. These observations suggest that the outer membrane cytochromes may, at least partially, be involved in UO _2_ formation. To better understand the role of S. oneidensis MR-1 outer membrane cytochromes (OMCs) in U(VI) reduction, we evaluated a mutant lacking all functionally active *c*-type cytochromes and constructed several mutants with targeted deletions of specific OMCs to evaluate their potential for extracellular reduction of U(VI). We compared the reduction kinetics of the cytochrome mutants with wild-type MR-1 resting cells and observed the differences in subcellular localizations of the UO _2_ nanoparticles in mutant strains after U(VI) reduction. Additionally, we used a novel combination of imaging and co-localization techniques to gain a better understanding of the organized extracellular UO _2_ nanoparticles and to gain insight into their biogenesis.

## Results

### Role of *c*-Type Cytochromes in Uranium Reduction

**Figure 1:**
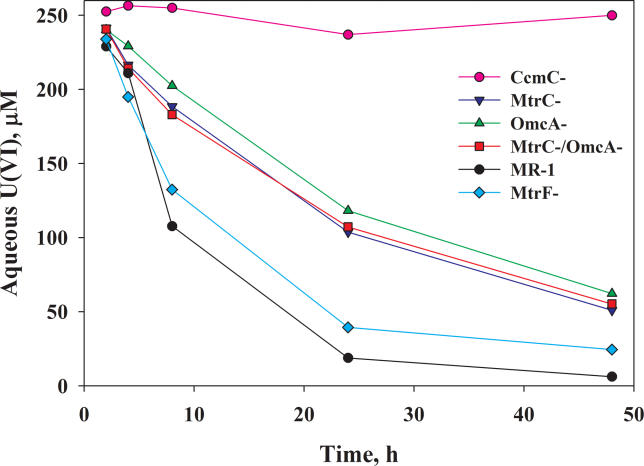
U(VI) Reduction Kinetics by S. oneidensis MR-1 and Cytochrome Mutant Cells The reduction of 250 μM U(VI) was determined for MR-1, a mutant lacking all *c*-type cytochromes (CcmC ^−^), single cytochrome deletion mutants (MtrC ^−^, OmcA ^−^, or MtrF ^−^), and a double cytochrome deletion mutant (MtrC ^−^/OmcA ^−^). Lines represent the mean data from representative experiments.

To investigate the importance of *c*-type cytochromes in U(VI) reduction, we used an S. oneidensis MR-1 mutant lacking the ability to covalently incorporate heme into nascent apocytochromes (CcmC ^−^) [ 26]. The CcmC ^−^ mutant was unable to reduce U(VI), present as uranyl carbonate complexes [ 27, 28], to U(IV) over a 48-h period, while wild-type MR-1 completely reduced 250 μM U(VI) ( *p* < 0.005) under identical conditions ( Figure 1). To further investigate the involvement of *c*-type cytochromes in U(VI) reduction, a series of OMC in-frame deletion mutants lacking either *mtrC*, *omcA*, *mtrF,* or both *mtrC* and *omcA* genes were constructed and verified by immunoblot analysis with specific sera ( Figure S1). In resting-cell reduction assays, the wild-type reduced U(VI) within 24 h, whereas MtrC ^−^, OmcA ^−^, and MtrC ^−^/OmcA ^−^ mutants reduced U(VI) at a slower rate, requiring 48 h to reduce approximately 200 μM U(VI) to U(IV) ( *p* = 0.001) ( Figure 1). In contrast, U(VI) reduction rates displayed by the MtrF ^−^ mutant were not significantly affected and were more similar to the wild-type than to the MtrC ^−^, OmcA ^−^, and MtrC ^−^/OmcA ^−^ mutants. While in-frame deletions of single or multiple OMCs slowed reduction rates, none of the mutants tested abolished the ability to reduce U(VI) as was seen with the CcmC ^−^ mutant.

The in vitro ability of purified OMCs to transfer electrons to U(VI) was tested and compared with the ability to reduce Fe(III)-NTA. Both reduced MtrC or OmcA were oxidized by Fe(III)-NTA within 2.5 s of exposure and both reactions were biphasic and followed first-order kinetics. Although purified MtrC was also oxidized by uranyl citrate ( *K _obs1_* = 0.039 ± 0.001 and *K _obs2_* = 0.008 ± 0.001) ( Figure S2), the biphasic reaction was not completed within 40 s and the reaction rate was more than 100 times slower than that of Fe(III)-NTA ( *K _obs1_* = 4.1 ± 0.13 and *K _obs2_* = 1.13 ± 0. 43). In contrast, reduced OmcA had no detectable electron transfer activity (< 0.5%) when reacted with uranyl citrate but was completely oxidized by Fe(III)-NTA ( *K _obs1_* = 2.96 ± 0.28 and *K _obs2_* = 0.9 ± 0.09). When equal amounts of OmcA and MtrC were combined, their electron transfer activity with uranyl citrate was similar to that observed with MtrC alone (unpublished data).

### Inactivation of OM *c*-Type Cytochromes Affects the Localization of UO _2_ Nanoparticles in S. oneidensis MR-1

**Figure 2:**
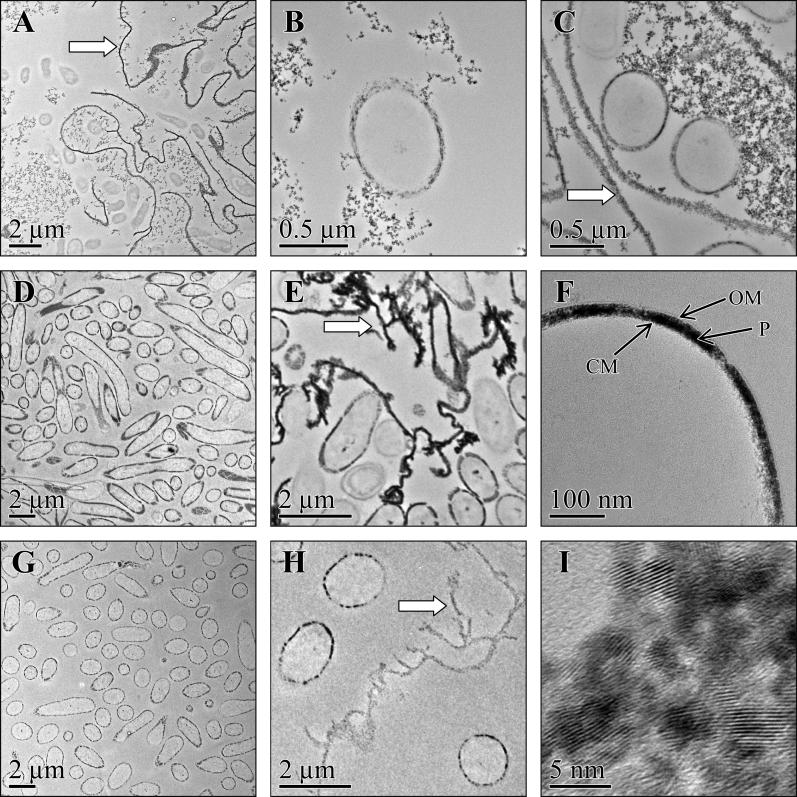
UO _2_ Localization in S. oneidensis MR-1 Wild-type and Cytochrome-Deficient Mutants TEM images prepared from cell suspensions incubated with 250 μM uranyl acetate and 10 mM lactate for 24 h. The localization of UO _2_ by MR-1 (A, B) was compared to OmcA ^−^ (C), MtrC ^−^ (D–F), and MtrC ^−^/OmcA ^−^ (G, H). High-resolution image of extracellular UO _2_ nanoparticles showing d-lines values consistent to previous patterns of biogenic and synthetic UO _2_ (I). The UO _2_-EPS is designated by the arrows. Locations of the cell membrane (CM), periplasm (P), and outer membrane (OM) are shown.

The subcellular localization of UO _2_ in wild-type MR-1 and the OMC deletion mutants was determined by transmission electron microscope (TEM) analysis of samples collected 24 h after the addition of U(VI) and lactate ( Figure 2). Thin sections of MR-1 revealed that UO _2_ was predominantly accumulated in cell suspensions as 1- to 5-nm particles ( Figures 2A, 2B, and S3). These UO _2_ nanoparticles were present primarily in one of three forms: densely packed particles in association with an extracellular polymeric substance (UO _2_-EPS) ( Figure 2A, arrow), loosely packed aggregates of particles not in association with EPS but external to cells ( Figure 2A and 2B, extracellular aggregates of lower contrast), or, to a lesser degree, localized within the cell periplasm ( Figure S3). The electron-dense material observed in all samples, regardless of location or association, consisted of U nanoparticles with selected area diffraction patterns consistent with those reported for synthetic and biogenic UO _2_ ( Figures 2I and S4). Similar to the wild-type, the OmcA ^−^ mutant localized UO _2_ nanoparticles in the periplasm as well as extracellularly in association with organized EPS structures and random patches of less densely arranged aggregates ( Figure 2C). In contrast to the wild-type, the accumulation of UO _2_ in the MtrC ^−^ or MtrC ^−^/OmcA ^−^ mutants was predominantly periplasmic and, to a much lesser degree, extracellular in association with the EPS ( Figure 2D– 2H). The loosely arranged aggregates of UO _2_ were absent in both the MtrC ^−^ and MtrC ^−^/OmcA ^−^ mutants ( Figure 2D and 2E). Although all three of the OMC deletion mutants exhibited UO _2_ nanoparticles in association with EPS, there were distinct differences in the abundance, distribution, and density of the particles localized on the UO _2_-EPS, with the exception of OmcA ^−^, which was comparable to the wild-type. The MtrC ^−^ mutant UO _2_-EPS features were much less evident relative to the wild-type but, when observed, were associated with densely packed UO _2_ particles arranged in short branches. The UO _2_-EPS features from OmcA ^−^ most closely resembled the wild-type in abundance, density of particles, and the branching. The MtrC ^−^/OmcA ^−^ mutant exhibited the lowest abundance and density of UO _2_-EPS, although the morphology and branching pattern were similar to those of wild-type and OmcA ^−^ strains.

### UO _2_-EPS Features Are Co-localized with Fe and P

**Figure 3:**
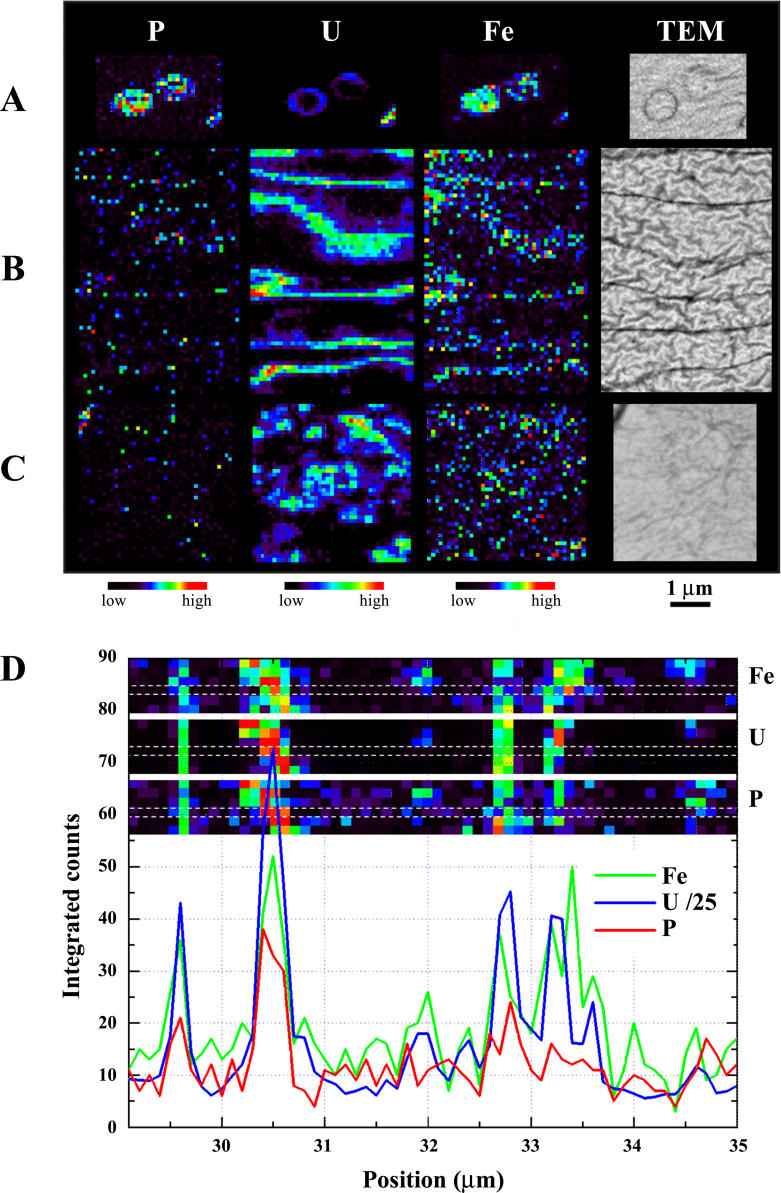
Synchrotron XRF Microscopy of the Elemental Concentration Gradients in Association with S. oneidensis MR-1 Cells False-color micro-XRF maps of qualitative spatial distributions and concentration gradients of P, U, and Fe in and around MR-1 cells. Cells are shown after incubation with 250 μM U(VI) for 24 h in standard assay conditions (A). The extracellular UO _2_ precipitates associated with EPS (B) and diffuse extracellular UO _2_ nanoparticles (C) observed in MR-1 samples were also evaluated for elemental composition. The scanned regions are represented with each corresponding thin-section TEM image. (D) The UO _2_-EPS features seen in (B) were scanned vertically six times longer per point, and the pixel intensity (identified between the dashed lines) was plotted for each element. Although this image is of a smaller area and has a smaller number of features in its field of view, the increased measurement time provides more robust statistics and further supports co-localization of the elements.

To obtain a better understanding of the features associated with UO _2_, the following samples analyzed by TEM were subjected to X-ray fluorescence (XRF) microscopy characterization: MR-1 cells, extracellular UO _2_ precipitates associated with EPS in MR-1 samples, and diffuse extracellular UO _2_ precipitates in MR-1 samples. False-color images of the P, U, and Fe fluorescence intensity for each sample type were aligned with the corresponding TEM image ( Figure 3A– 3C), and relative area concentrations of these elements in each location were calculated ( Figure S5). The shapes observed in the U fluorescence maps clearly corresponded to the morphology observed by TEM, with the highest concentration of P and Fe in MR-1 cells, and was consistent with other studies [ 29]. This was most evident in the UO _2_-EPS, where a high resolution scan (6-fold longer) of U, Fe, and P concentrations illustrated the spatial co-localization of these elements ( Figure 3D). The detection of both P and Fe in the UO _2_-EPS provided additional evidence for the bacterial origin of these structures, while the P and Fe distributions found within the diffuse UO _2_ aggregates appeared more or less randomly.

**Figure 4:**
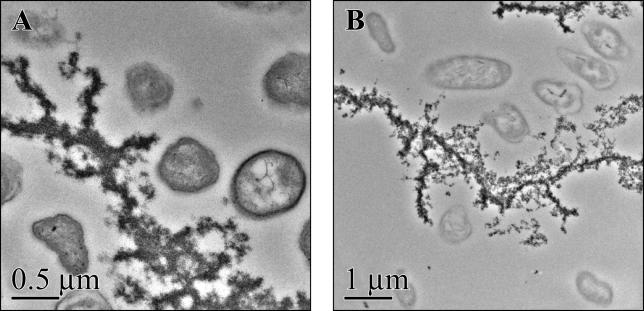
Heme Staining of Extracellular Cytochromes from S. oneidensis MR-1 TEM images of thin sections of MR-1 incubated for 24 h with 100 μM U(VI) and stained for the presence of heme. Samples were incubated with DAB and developed with H _2_O _2_ (A) or with DAB but not developed with H _2_O _2_ (B) prior to embedding. Heme-containing proteins detected in (A) were shown in close association with the undeveloped UO _2_-EPS seen in (B).

Heme staining was used to ascertain that the Fe signal in the UO _2_-EPS detected by XRF was indicative of heme-containing metalloprotein(s). MR-1 cells incubated in the presence of U(VI) showed heme-bound peroxidase activity which was uniformly distributed throughout the UO _2_-EPS ( Figure 4A). Moreover, when a similarly prepared sample was reacted with diaminobenzidine (DAB) but not developed with H _2_O _2_, the UO _2_ nanoparticles were observed in the EPS material, but heme-bound peroxidase activity was not detected ( Figure 4B). Together, this suggested that heme-containing proteins were in close association with the UO _2_-EPS. It is of interest to note that the H _2_O _2_ used to develop the DAB stain caused partial oxidation of UO _2_; however, the localization of the heme-containing proteins and the UO _2_-EPS was still apparent.

**Figure 5:**
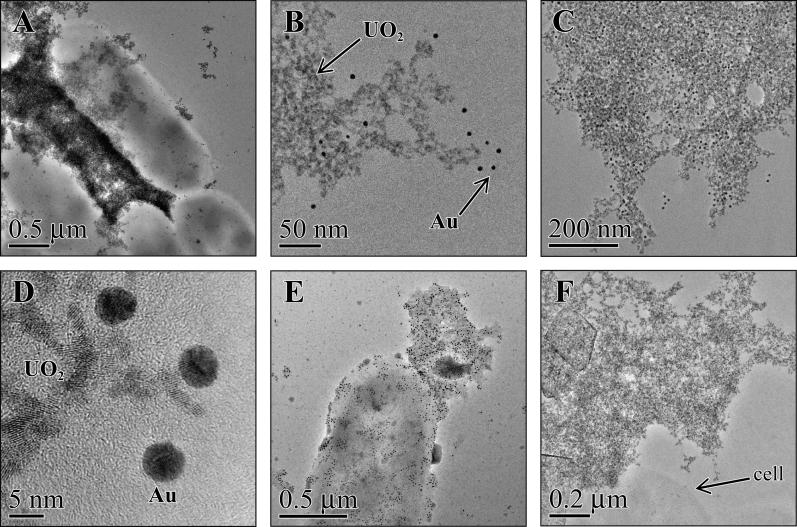
Immune-Localization of MtrC, OmcA, and MtrB with Extracellular UO _2_ from S. oneidensis MR-1 Whole-mount TEM images of MR-1 incubated with 100 μM U(VI) for 24 h and reacted with specific antibodies to MtrC (A, B), OmcA (C, D), or MtrB (E). High-resolution image of nanocrystalline UO _2_ associated with the extracellular matrix and the 5-nm particles of colloidal gold (Au) (B, D). Extracellular matrix and cell labeled with colloidal gold in the absence of specific antibody (F).

Using high-resolution immune-TEM, the localization of the OMCs in relation to the extracellular UO _2_ matrix was investigated ( Figure 5). Polyclonal antibodies which were produced toward unique surface-exposed domains of OmcA and MtrC revealed that these proteins were in close proximity with the cell-free UO _2_-EPS matrix ( Figure 5B and 5D) and were rarely observed in association with cell surfaces ( Figure 5A). MtrC and OmcA were consistently co-localized with each other and the UO _2_ nanoparticles. Samples not receiving MtrC- or OmcA-specific antibody did not reveal any labeling by colloidal gold of either the extracellular matrix or the cell surface ( Figure 5F). Interestingly, immune-TEM revealed the close association of the integral OM protein MtrB with extracellular UO _2_-EPS matrix ( Figure 5E). MtrB was also densely distributed over exposed regions of the MR-1 cell surface.

### The UO _2_-EPS Is a Complex Glycocalyx-Like Structure

**Figure 6:**
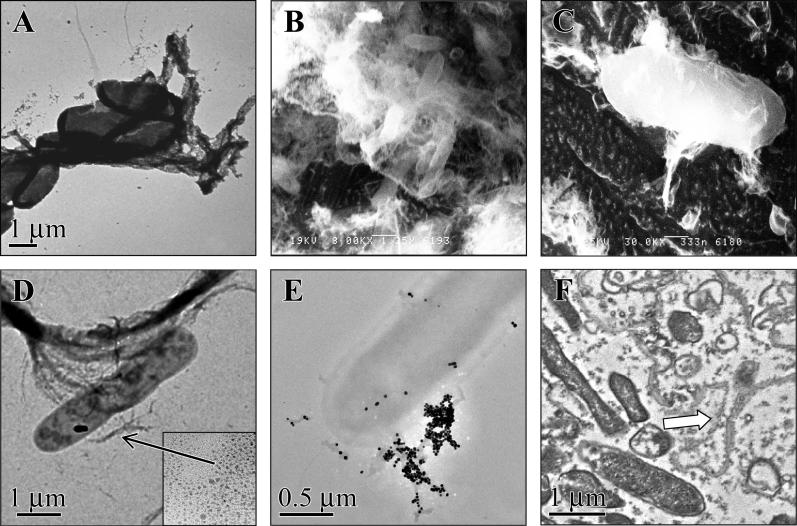
Extracellular Structure of S. oneidensis MR-1 Whole mounts of MR-1 incubated with 250 μM U(VI) for 24 h prior to visualization by TEM (A) or incubated in defined media and visualized by cryo-HRSEM (B, C). Whole-mount TEM of cells incubated with 1 mM fumarate added as the electron acceptor in place of U(VI) and reacted with positive charged colloidal nanogold particles (D) to help determine surface charge of the EPS matrix or the glycoconjugate-specific lectin, ConA, complexed with 40-nm particles of colloidal gold (E). High-resolution image of 1.4-nm gold nanoparticles (D inset). Thin-section TEM images of MR-1 incubated for 24 h with 1 mM fumarate prior to ruthenium red staining to visualize extracellular EPS (F). The ruthenium red-EPS is designated by the arrow.

To further investigate the structure of UO _2_-EPS matrix, resting cells of S. oneidensis MR-1 were incubated in the presence of 250 μM U(VI) without shaking to minimize shear forces. The UO _2_-EPS visualized by whole-mount TEM appeared around many cells ( Figure 6A) and also contained features which we attribute to the dehydration and collapse of an extracellular matrix similar to that observed using conventional fixation methods [ 30]. The use of cryo-HRSEM to preserve the complex three-dimensional structure eliminated the dehydration artifact observed in fixed U(VI)-reducing cultures. When samples were grown anaerobically in defined medium and prepared for cryo-HRSEM, the EPS matrix appeared as an intricate three-dimensional structure encompassing multiple cells. The visualization of single MR-1 cells demonstrated the delicate morphology of this material ( Figure 6B and 6C). This demonstrated that the EPS was not only associated with resting cell suspension incubated with lactate and U but was produced under growth conditions.

Furthermore, insight into the composition of the extra-cellular matrix was gained using electrostatic charge determination, glycoconjugate-specific staining, and glycocalyx fixation techniques. In the absence of UO _2_, either positively or negatively charged particles were used to probe the charge characteristics of the extracellular material. Cationic nanogold particles were bound to small patches near the cell surface and to the EPS matrix ( Figure 6D). Binding of anionic nanogold particles was not observed in similarly prepared samples. The glycoconjugate component of the extracellular EPS matrix was visualized using a lectin-colloidal gold complex. These samples showed an amorphous EPS matrix that was densely labeled with the gold ( Figure 6E). These structures were similar to the extracellular UO _2_-EPS matrix observed during immune-TEM analysis of MtrC and OmcA. The glycoconjugate affinity was rarely observed in association with cell surfaces. These findings indicated that the UO _2_-EPS matrix also contained a significant glycoconjugate fraction.

Ruthenium red-lysine fixation was chosen as both a fixative and a stain to provide added stability and contrast to elaborate extracellular structures surrounding wild-type MR-1 cells grown in the absence of UO _2_. Ultrastructural analysis of samples fixed with this technique displayed an extended EPS that appeared electron dense due to the interaction with the ruthenium red ( Figures 6F and S6). No staining with osmium tetroxide, uranyl acetate, or lead citrate was required to visualize these features. These extracellular structures were morphologically identical to the heme-containing EPS with a high density of associated UO _2_ particles observed after U(VI) reduction by MR-1.

## Discussion

The widespread distribution, metabolic versatility, and ability to respire metals as terminal electron acceptors underscore the important ecological role of *Shewanella* species in metal cycling in natural environments and their potential importance in controlling reductive transformation processes and metal mobility in contaminated groundwater. Earlier studies using Shewanella sp., Geobacter sp., and Desulfovibrio sp. demonstrated both extracellular and/or periplasmic accumulation of reduced UO _2_ particles and suggested that this process has important implications for the immobilization of U [ 5, 18, 25, 31– 33]. Previous investigations, however, did not identify the mediators of U(VI) reduction or the genesis of materials associated with the extracellular UO _2_. To address these questions, we used a novel combination of genetic, immunological, and microscopic analyses including targeted gene deletion, high-resolution microscopy, synchrotron-based XRF microscopy, heme staining of noncellular structures intricately associated with UO _2_, and visualization of the metal oxide-cytochrome interaction by high resolution immune-localization.

In this study, we established that MtrC, a decaheme *c*-type cytochrome previously reported to be involved in Fe(III) and Mn(IV) reduction [ 8, 26], is responsible for at least a portion of the total extracellular U(VI) reductase activity in S. oneidensis MR-1. We found that deletions of *mtrC* or both *mtrC* and *omcA* genes significantly slowed the rate of reduction of U(VI) and affected the distribution and density of the U(IV) particles localized on the extracellular features. Our findings are in agreement with a recent report [ 34] that the absence of MtrC did not abolish but significantly decreased the reduction of U(VI) in MR-1. Interestingly, the deletion of another OM decaheme *c*-type cytochrome, *mtrF,* had little impact on the rate of U(VI) reduction. Although the amino acid homology of MtrF with MtrC (approximately 38%) suggested a similar function, to date there have been no reports of the involvement of MtrF in electron transfer to metals. Using in vitro electron transfer assays with recombinant cytochromes exhibiting Fe(III)-reductase activity, we demonstrated that MtrC, but not OmcA, can function as a terminal reductase of uranium. The in vivo experiments suggest that OmcA affected the rate of U(VI) reduction similar to MtrC and thus was important for U(VI) reduction and electron transfer. Since the in vitro mixture of MtrC and OmcA did not enhance electron transfer rates, the native system may also require additional, as-of-yet-undetermined protein(s).

Moreover, we demonstrated that a mutant of S. oneidensis MR-1 deficient in cytochrome *c* maturation is unable to reduce soluble U(VI) carbonate complexes, indicating that functional *c*-type cytochromes are essential for U(VI) reduction and that MR-1 lacks a secondary independent U(VI) reductase system. Although this observation does not unequivocally rule out the involvement of specific redox enzymes in U reduction, we believe that reductive precipitation of U(IV) in S. oneidensis MR-1 is a process driven by low-potential periplasmic or OM-associated *c*-type cytochromes. Given the large number of predicted periplasmic and cytoplasmic membrane *c*-type cytochromes in the MR-1 genome [ 7] coupled with their typical lack of specificity in regards to electron transfer to metal ions, it seems likely that many of these low-potential *c*-type cytochromes may be capable of transferring electrons to U(VI) within the periplasm. We hypothesize that a complex network of *c*-type cytochromes with some functional redundancy, including MtrC, other OMCs, as well as periplasmic cytochromes, can function as uranyl reductases and influence the localization of both periplasmic and extracellular UO _2_ nanoparticles in resting cell suspensions of S. oneidensis MR-1. The involvement of a redundant network of both OM and periplasmic cytochromes for U(VI) reduction has not previously been reported for *Shewanella* or other U(VI)-reducing bacteria. Biochemical studies suggest that low-molecular-mass *c*_3_ or *c*_7_ cytochromes located in the periplasm are important electron carriers in U(VI) reduction by Desulfovibrio sp. and Geobacter sp., respectively [ 11, 35]. Interestingly, Lloyd et al. found that the periplasmic *c*_7_ cytochrome PpcA, produced by *Geobacter sulfurreducens,* was not the sole U(VI) reductase [ 11] but also reported that the surface OMCs are not involved in U(VI) reduction [ 31]. Clearly, further studies will be required to fully understand the complete electron transfer pathways involved in microbial U(VI) reduction.

The combination of high-resolution imaging, XRF microscopy, and immune-localization analyses used in this study support the biological origin of the EPS material containing dense accumulations of UO _2_ nanoparticles. We established that the extracellular U(IV) nanoparticles are in close association with the MtrC and OmcA decaheme *c*-type cytochromes which are present within the EPS. While the direct involvement of MtrC in U(VI) reduction is not surprising, this is the first report of extracellular localization of a decaheme cytochrome in direct association with UO _2_ nanoparticles. It has recently been reported that MtrC and OmcA form a functional high-affinity complex in vivo [ 36]. This finding would explain the co-localization of MtrC and OmcA in direct association with the UO _2_ nanoparticles, although the latter had very little effect on the localization of UO _2_ nanoparticles and was unable to function as a terminal reductase of U(VI) citrate in vitro.

Significantly, the presence of an integral OM protein (MtrB) within the UO _2_-EPS matrix as well as on the cell surface of MR-1 suggests that the extracellular material may be comprised, at least in part, of OM or an OM-derived material. MtrB has previously been shown to have epitopes exposed on the outside surface of the S. oneidensis MR-1 OM and has not been found in soluble cell extracts [ 17]. Together, this evidence suggests the existence of an OM-like EPS produced by MR-1 associated with high-molecular decaheme *c*-type cytochromes which promote the formation of biogenic UO _2_ nanoparticles.

In some gram-negative bacteria, such as Pseudomonas putida G7, EPS has been shown to have a significant metal-binding capacity [ 30]. Since our findings suggested that the matrix was negatively charged, we hypothesized that electrostatic interactions may have been involved in the formation of the UO _2_-EPS structures in S. oneidensis MR-1. Olsson et al. [ 37], reported that the surface charge of UO _2_ (pH of point of zero charge = 5.0 to 5.5) at similar pH conditions would also be negative and thus electrostatic interactions may not be responsible for binding of the biogenic UO _2_ nanoparticles. However, these same authors note that the oxidation of the UO _2_ surface can lead to higher point of zero charge values, and such effects cannot be excluded here. Given the complexity of the extracellular matrix including the *c*-type cytochromes OmcA and MtrC, other undetermined factors may also attribute to the strong interaction of the matrix with the UO _2_. These interactions could be advantageous to maintaining nanoparticle stability because the individual fine-grained UO _2_ particles observed in this study were of a size (1 to 5 nm) that would be subject to rapid reoxidation by O _2_ [ 38] or colloidal transport. The apparently close, interactive molecular association of the nanoparticulate UO _2(s)_ with these complex biopolymers in the environment could influence (e.g., slow) the oxidation rate of U(IV) and prevent the mobilization of the small precipitates as dispersed colloids in pore or groundwater. Collectively, our results imply that the environmental behavior of the biogenic UO _2(s)_ will be strongly influenced by this unusual structural association.

Recent microarray expression studies have shown that approximately 7% of all MR-1 genes upregulated under U(VI)-reducing conditions encode proteins involved in membrane/periplasmic stress response [ 34]. Unlike chromium(VI), there does not appear to be a U(VI)-specific detoxification system in MR-1 [ 34]. This finding could possibly explain the formation of the UO _2_-EPS as a turnover mechanism to rid cells of UO _2_. While the detailed composition and genesis of the material associated with the UO _2_-EPS remain undetermined, the presence of the lipoproteins MtrC and OmcA, integral OM protein, and the glycoconjugate component together suggests that multiple elements of the OM and polysaccharide are key components of these structures. The formation of the UO _2_-EPS matrix observed in our study may represent an important mechanism by which *Shewanella* is able to rid the cell periplasm and surface of the UO _2_ nanoparticles that are clearly generated from more than one *c*-type cytochrome. Alternatively, the EPS produced by *Shewanella* may be an extension of the OM-bound electron transport chain that is directly involved in extracellular U(VI) to UO _2_ nanoparticle formation that remains in association with EPS [ 33, 39– 41].

Although its exact function remains to be determined, production of EPS by S. oneidensis MR-1 does not appear to be required for U(VI) reduction since OMC mutants that produce little UO _2_-EPS are capable of reducing U(VI). Studies are under way to isolate mutants with reduced or abolished ability to produce EPS and to characterize their impact on U(VI) reduction and localization as well as to determine whether extracellular UO _2_ nanoparticles observed in association with other U(VI)-reducing bacteria are similarly associated with EPS [ 18, 31– 33].

This report is the first to confirm the role of *c*-type cytochromes in the reduction of U(VI) in S. oneidensis MR-1 and, more specifically, directly link OM-associated *c*-type cytochromes with U(VI) reduction and localization outside the cell. Furthermore, we conclusively show the intimate association of these high-molecular cytochromes with extra-cellular biogenically reduced UO _2_. While the exact function(s) of this novel cytochrome-UO _2_ association remains unclear, this co-localization could have important implications for understanding long-term fate of biogenic UO _2_ in subsurface environments.

## Materials and Methods

### Chemicals and media.

All chemicals used in this study were purchased from Sigma Chemical Co. (St. Louis, Missouri, United States) unless otherwise noted. Growth media were purchased from BD Diagnostics (Sparks, Maryland, United States).

### Generation of cytochrome deletion mutants.

S. oneidensis MR-1 mutants lacking selected OMC genes were constructed using two-step homologous recombination with a suicide plasmid encoding flanking DNA sequence with a modification of previously described methods [ 42, 43]. The detailed procedures outlining mutant construction and the primers, plasmids, and strains used in this study are described in detail in Protocol S1 and Tables S1 and S2.

### U(VI) reduction and localization assay conditions.

The kinetics of aqueous U(VI) reduction and localization in wild-type MR-1 and mutant cells were determined in a standard resting cell assay. Tryptic soy broth–dextrose cultures (100 ml) were grown for 16 h (30 °C) at 100 rpm and harvested by centrifugation (5,000 × *g*, 5 min). Cells were washed once in equal volume of 30 mM sodium bicarbonate buffer (pH 7.0, 4 °C), pelleted, and standardized by suspending all treatments in the fresh buffer at a concentration of 2 × 10 ^9^ cells/ml prior to being purged for approximately 10 min with mixed gas (N _2_/CO _2_ 80:20). U(VI) reduction assays contained a final concentration of 250 μM U(VI) as uranyl acetate and 10 mM sodium lactate in 30 mM sodium bicarbonate purged with O _2_-free mixed gas and sealed with thick butyl rubber stoppers. Kinetic studies were initiated by the addition of 1 ml of standardized cells to the assay tubes followed by horizontal incubation at 30 °C with slow gyratory shaking (25 rpm) resulting in a final assay density of 2 × 10 ^8^ cells/ml. The amount of soluble U(VI) remaining in filtrates (less than 0.2-μm pore size) from all samples was analyzed at multiple time points using a kinetic phosphorescence analyzer (KPA-10; Chemchek Instruments, Richland, Washington, United States) as previously described [ 44]. Metal reduction curves were compared using nonparametric procedures, specifically the Wilcoxon signed-rank test. These tests were conducted using Systat 10 (SPSS Inc, Chicago, Illinois, United States) and were considered significant at *p* < 0.01; specific values of *P* are reported where relevant.

### Reductase activity of recombinant cytochromes.

The recombinant *c*-type cytochromes, OmcA and MtrC, were expressed and purified as described previously [ 36]. Proteins were prepared at a concentration of 10 μM (100 μM heme) in buffer containing 100 mM HEPES buffer (pH 7.5), 50 mM NaCl, 10% glycerol, and 1% (w/v) of *n*-octyl-β- d-glucopyranoside and purged with O _2_-free N _2_ gas. The reaction of recombinant cytochrome (rMtrC, rOmcA, or rMtrC and rOmcA), reduced by titrating with dithionite, with U(VI) was initiated by the addition of equal volumes of cytochrome with 300 μM U(VI) in 5 mM sodium citrate buffer in an anoxic atmosphere. Oxidation of heme was monitored using a Hi-Tech SFA-20 stopped-flow system with a 1-cm pathlength cell integrated with a Hewlett-Packard 8543 diode-array spectrophotometer capable of following reaction kinetics at multiple wavelengths. To access the activity of the purified cytochromes used for U(VI) experiments, the reaction of dithionite-reduced cytochrome with an equal volume of 300 μM Fe(III)-NTA in 100 mM HEPES buffer (pH 7.5) was also monitored. The reaction between reduced cytochrome and either U(VI) or Fe(III)-NTA was analyzed using protocols detailed by Dobbin et al. [ 45].

### Production of antibodies.

Affinity-purified antibodies toward predicted hydrophilic and surface-exposed regions of MtrC, OmcA, and MtrB were designed and produced commercially (Biosynthesis, Lewisville, Texas, United States) ( Table S3). The peptide sequences selected for antibody production were confirmed for antigenic uniqueness using BLASTP analysis against all MR-1 proteins. Affinity purified antibodies, from 0.4 to 0.7 mg/ml stocks solutions, were tested for specificity using immunoblots of MR-1 and mutant cells as described in Protocol S1.

### TEM.

Cells were prepared for TEM of plastic sections in an anaerobic glove bag (Ar/H _2_, 95:5) using anoxic solutions. Three milliliters of cell suspension incubated for 24 h with U was centrifuged (2,300 × *g*, 5 min), and the cell pellet was fixed in 2.5% glutaraldehyde (Electron Microscopy Sciences [EMS], Fort Washington, Pennsylvania, United States) prior to dehydration in an ascending series of ethanol and infiltration in LR White embedding resin (EMS) and cured at 60 °C. Blocks were sectioned anaerobically to 70 nm with a Diatome 45-degree diamond knife using an Ultracut UCT ultramicrotome (Leica, Bannockburn, Illinois, United States) and mounted on 200 mesh copper grids with formvar support film coated with carbon. Unstained sections were examined at 200 kV using JEOL 2010 high-resolution TEM equipped with LaB _6_ filament with a resolution of 1.9 Å. Images were digitally collected and analyzed using DigitalMicrograph software (Gatan Inc, Pleasanton, California, United States). The elemental composition of precipitates was determined using electron dispersive spectroscopy (Oxford Instruments, Fremont, California, United States) equipped with SiLi detector and analyzed with ISIS software. Selected area diffraction patterns were evaluated using the Desktop Microscopist software (Lacuna Laboratories, Tempe, Arizona, United States).

### Cryo-high-resolution scanning electron microscopy.

Samples of wild-type MR-1 were grown anaerobically with fumarate in modified basal minimal medium (pH 7.5) [ 46] without agitation and prepared for cryo-high-resolution scanning electron microscopy (HRSEM) as described by Apkarian et al. [ 47]. Bacterial cell suspension was frozen in high-pressure freezer (Bal-Tec), transferred onto a cryostage (Oxford CT-3500), and sputtered with chromium. Samples were examined at in-lens cryo-HRSEM (DS-130F) at 25 kV at −150 °C. Imaging was done with minimal dwell time to eliminate the beam damage, resulting in images of fully hydrated, unfixed specimens immersed in featureless amorphous ice.

### Characterization of extracellular matrix by TEM.

For immune-localizations, cells were prepared as described above except that a final concentration of 100 μM U(VI) was used. After 24-h incubation, cells were briefly fixed in 2% paraformaldehyde (EMS) and 0.1% glutaraldehyde. Following fixation, whole mounts were prepared by placing 10 μl on formvar/copper grids and the liquid removed by wicking. Whole mount TEM grids were also prepared in a similar manner on unfixed cells incubated with 250 μM U(VI) without shaking. Immune-localization samples were blocked in PBS (10 mM sodium phosphate [pH 7.2] and 140 mM sodium chloride) containing 2% BSA (PBS/BSA). Antibodies (diluted 1:2 in PBS/BSA) were reacted for 30 min with the samples followed by five PBS washes before incubation with the 5-nm gold secondary antibody (diluted 1:5 in PBS/BSA). Samples were washed five times in PBS and fixed with 2.5% glutaraldehyde followed by two water rinses. Antibody specificity was verified in all localization studies by reacting similarly prepared grids with colloidal gold detection antibody in the absence of specific antibody and by using naïve sera as controls.

The detection of heme by TEM was performed using 3,3′-DAB (EMS) [ 48]. Cells were collected by centrifugation and fixed for plastic embedding as described above. The fixative was replaced by three washes in 100 mM sodium cacodylate buffer (EMS) followed by three incubations (two 15-min and one 10-min) in cacodylate buffer containing fresh DAB. The heme stain was developed by the addition of 600 μl of fresh DAB solution and 30 μl of 3% H _2_O _2_. Control samples received fresh DAB solution without 3% H _2_O _2_. The reaction was stopped by washing three times in cacodylate buffer before embedding.

The extracellular matrix was investigated in the absence of U by labeling with charged nanogold particles, glycoconjugate visualization, or ruthenium red staining. Cells were prepared as described above except that 1 mM fumarate was added as the electron acceptor in place of U(VI). To determine the surface charge of the extracellular material, whole mounts were prepared for TEM and reacted with either positive or negative charged nanogold particles (1.4 nm) (Nanoprobes, Yaphank, New York, United States). Grids were labeled for 1 min with nanogold particles diluted 1:5 in cacodylate buffer followed by one rinse in cacodylate buffer and two water rinses. For glycoconjugate visualization, samples were reacted with a lectin, concanavalin A, conjugated with 40-nm gold beads (EY Laboratories, Inc, San Mateo, California, United States) diluted 1:5 in cacodylate buffer for 2 min. Samples were rinsed twice in cacodylate buffer followed by two water rinses. To visualize delicate extracellular structures such as a glycocalyx, a ruthenium red-lysine fixation was chosen [ 49]. An equal volume of 2× stain cocktail (60 mM lysine, 4% paraformaldehyde, 5% glutaraldehyde, 0.15% ruthenium red, and 200 mM cacodylate buffer) was added to each sample and gently mixed by inversion. After 15 min, cells were collected by centrifugation at 2,300 × *g* for 30 s. Cells were washed three times in 100 mM cacodylate buffer followed by dehydration and embedding as described above.

### Synchrotron XRF analysis.

Synchrotron-based XRF microscopy analysis [ 29] was performed using the 2IDD beam line [ 50] at the Advanced Photon Source (Argonne, Illinois, United States). The steps involved in beam-line calibration, generation of two-dimensional elemental maps, and XRF spectrum analysis are described in Protocol S1.

## Supporting Information

Figure S1 Immunoblot Analysis of the MtrC/OmcA Cytochromes in S. oneidensis MR-1 and Cytochrome Mutants
Immunoblot analysis of 10 μg of total protein from overnight cultures of MR-1 (lanes 1), MtrC ^−^ (lanes 2), OmcA ^−^ (lanes 3), and MtrC ^−^/OmcA ^−^ (lanes 4) resolved by SDS-PAGE and developed with specific antibodies toward MtrC (A) or OmcA (B).
(2.0 MB TIF)

Figure S2 Oxidation Rates of Reduced MtrC by Uranium Citrate The oxidation of dithionite-reduced 10 μM MtrC in HEPES buffer (pH 7.5) was calculated when mixed with 300 μM U(VI) in sodium citrate buffer. The oxidation of heme was monitored in an anoxic atmosphere.
(1.2 MB TIF)

Figure S3 UO _2_ Localization in S. oneidensis MR-1 Cells
TEM micrographs prepared from cell suspensions incubated with 250 μM uranyl acetate and 10 mM lactate for 24 h. The localization of the UO _2_-EPS in close association with MR-1 cells (A–C). High-resolution images of cells illustrate the localization of UO _2_ relative to the outer and cell membranes of intact cells (C–F). The UO _2_-EPS is designated by the arrows. Locations of the cell membrane (CM), periplasm (P), and OM are shown.
(7.0 MB TIF)

Figure S4 TEM-Coupled Analysis of Extracellular UO _2_ Nanoparticles
Nanocrystalline UO _2_ material was evaluated by selected area electron diffraction (A) and electron dispersive spectrometry (B).
(6.3 MB TIF)

Figure S5 Quantification of the Elemental Area Concentrations within Structures Studied by XRF Analysis The counts under the peaks of each element in the background-subtracted spectra were used to determine area concentrations of Fe (A) and P (B) in each object of interest. Volume concentrations (ppm) were obtained by assuming a uniform 110 nm thickness of the slices, density of 1.0 g/cm ^3^, uniform coverage of material within the dimension of the X-ray probe, and a uniform distribution along the sample thickness. Error bars in the final concentrations account only for sample-to-sample variability in the final concentrations.
(582 KB TIF)

Figure S6 Ruthenium Red Staining of Extracellular Structures from S. oneidensis MR-1
Thin section TEM images of MR-1 incubated for 24 h with 1 mM fumarate prior to ruthenium red staining to visualize extracellular structures. The ruthenium red-associated EPS is designated by the arrows.
(3.4 MB TIF)

Protocol S1 Supporting Methods (44 KB DOC)

Table S1 Bacterial Strains and Plasmids Used for This Study (26 KB DOC)

Table S2 Primers Used to Create the In-frame Mutants in This Study (19 KB DOC)

Table S3 Peptide Sequences Used to Produce Specific Antisera (27 KB DOC)

### Accession Numbers

The GenBank ( http://www.ncbi.nlm.nih.gov/Genbank) accession numbers for the protein sequences described in this paper are found are MtrC (gi|24373344), OmcA (gi|24373345), and MtrF (gi|24373346) *.*

## Abbreviations

DAB: diaminobenzidine
DMRB: dissimilatory metal-reducing bacteria
EPS: extracellular polymeric substance
HRSEM: high-resolution scanning electron microscopy
OM: outer membrane
OMC: outer membrane cytochrome
TEM: transmission electron microscope
U: uranium
UO _2_: uraninite
XRF: X-ray fluorescence
