# Supplementary material for: c-Type Cytochrome-Dependent Formation of U(IV) Nanoparticles by Shewanella oneidensis
Source: PLoS Biol. 2006 Aug 8;4(8):e268. doi: 10.1371/journal.pbio.0040268 (PMC1526764; doi:10.1371/journal.pbio.0040268)
Supplement: Supplementary file 1 [file pbio.0040268.sd001.doc]

**Protocol S1**

***c*-Type Cytochrome-Dependent Formation of U(IV) Nanoparticles by *Shewanella oneidensis***

Matthew J. Marshall^1^, Alexander S. Beliaev^1*^, Alice C. Dohnalkova^1^, David W. Kennedy^1^, Liang Shi^1^, Zheming Wang^2^, Maxim I. Boyanov^3^, Barry Lai^4^, Kenneth M. Kemner^3^, Jeffrey S. McLean^1^, Samantha B. Reed^1^, David E. Culley^1^, Vanessa L. Bailey^1^, Cody J. Simonson^1^, Daad A. Saffarini^5^, Margaret F. Romine^1^, John M. Zachara^2^, and James K. Fredrickson^1*^

^1^Biological Sciences Division, Pacific Northwest National Laboratory, Richland, Washington 99354, USA

^2^Chemical Sciences Division, Pacific Northwest National Laboratory, Richland, Washington 99354, USA

^3^Biosciences Division, Argonne National Laboratory, Argonne, Illinois 60439–4843, USA

^4^Advanced Photon Source, Argonne National Laboratory, Argonne, Illinois 60439–4843, USA

^5^Department of Biological Sciences, University of Wisconsin, Milwaukee, Wisconsin 53211, USA

*** Corresponding Author Information:** Correspondence and requests for materials should be addressed to J.K.F (jim.fredrickson@pnl.gov) and A.S.B. (alex.beliaev@pnl.gov) at Pacific Northwest National Laboratory, MSIN P7-54, P.O. Box 999, Richland, WA 99354. Phone: (509) 376-7063. Fax: (509) 376-4909.

**Generation of In-Frame Deletion Mutants.** *Shewanella oneidensis* MR-1 and *Escherichia coli* strains used for mutagenesis were grown using Luria-Bertani (LB) medium at 30°C and 37°C, respectively. The primers, plasmids, and strains used in this study are described in Tables S1 and S2. The plasmid pDS3.1 was purified from *E. coli* using the Qiagen QIAprep Spin Miniprep Kit (Qiagen, Valencia, CA) and digested with *Xcm*I (New England Biolabs (NEB), Beverly, MA). *S. oneidensis* chromosomal DNA was isolated using DNAzol (Invitrogen, Carlsbad, CA) and used as the template for PCR amplification using Vent DNA polymerase (NEB). Sequences that flank the respective genes targeted for deletion were joined by amplifying each locus with 5-O/5-I and 3-O/3-I primer pairs, annealing them via complementary sequences present in the 5-I and 3-I primers, and then subjected to a second round of PCR using the 5-O and 3-O primers as described [1]. The fusion PCR amplicon was purified using 1% agarose electrophoresis [2] and ligated into the pDS3.1 plasmid using the FastLink Ligation kit (Epicentre, Madison, WI) prior to transformation into *E. coil* EC100D pir-116 (Epicentre) [3] and plating on LB with gentamycin selection (15 μg/ml). Correct transformants were verified by PCR using the 5-O and 3-O primers and the plasmids were purified and transformed into the *E. coli* ß-2155 mating strain by plating on gentamycin (15 μg/ml) and diaminopimelic acid (100 μg/ml) selection. Conjugal transfer of plasmid and homologous recombination from *E. coli* mating strain to *S. oneidensis* was performed [4] and primary integrants were selected with gentamycin (7.5 μg/ml). PCR screening for homologous recombination of the plasmid and the insertion site of amplicon recombination within the genome were accomplished using the F-O/3-O or 5-O/R-O primer pairs. Sucrose sensitivity of the primary integrants was verified by plating on LB-NaCl +10% sucrose [5]. The second homologous recombination was selected for by growing the primary integrant for 16-18 hours in LB-NaCl broth cultures followed by plating on LB-NaCl +10% sucrose. Colonies sensitive to gentamycin (7.5 μg/ml) were screened for deletion of the gene of interest by PCR using the F-O/R-O primer set and compared to the same fragment amplified from wild type MR-1. The PCR amplicon generated form the mutants with the F-O/R-O primer set was used as the template for DNA sequencing of the deleted gene(s) and recombination regions (ACGT, Inc., Wheeling, IL). Protein expression was verified by resolving 10 μg total protein from overnight TSB -dextrose cultures (16 hours) on SDS-PAGE (4-20% Tris-glycine gels, Invitrogen) followed by immunoblotting with specific antisera. Immunoblots were visualized using anti-rabbit specific secondary antibodies and Western Blue substrate for alkaline phosphopatase (Promega, Madison, WI).

**Synchrotron X-Ray Fluorescence Analysis.** Synchrotron-based X-ray fluorescence (XRF) microscopy analysis was performed on sections (110 nm) from samples incubated with U. Sections were mounted on nickel grids with formvar support coated with carbon and transported to the 2IDD beamline [6] at the Advanced Photon Source (Argonne, IL) for analysis. The incident beam energy was tuned to 7300 eV, with beam size of 150 nm (FWHM) at the focal point. The samples were raster-scanned in front of the beam in 100 nm steps and the XRF emission spectrum from each point was recorded using a single element Ge detector and a multi-channel analyzer (MCA). Energy calibration of the MCA and quantification of elemental concentrations were accomplished using thin film glass standards (National Institute of Standards and Technology (NIST) 1832 and 1833). Two-dimensional elemental maps of the samples were obtained using the unprocessed XRF spectra collected with the MCA at each spatial position, by integrating the K_a_ XRF signal of Si, P, and Fe, and the M_a_ XRF signal of U. The objects of interest were identified by comparison to the TEM contrast images. The spectra from regions within the objects were averaged to produce a representative XRF spectrum for each sample. Background was removed from each sample’s spectrum by subtracting from it (1) the averaged spectrum from a region close to, but not containing the object, (2) an XRF spectrum from UO_2_, scaled to the U XRF intensity of the sample, and (3) a Si K_a_ XRF signal, scaled to the intensity of the Si signal in the sample. Procedures (2) and (3) remove the large backgrounds produced by these two elements and allow for reliable spectral comparisons between different samples.

**References**

1. Link AJ, Phillips D, Church GM (1997) Methods for generating precise deletions and insertions in the genome of wild-type *Escherichia coli*: Application to open reading frame characterization. J Bacteriol 179: 6228-6237.

2. Sambrook J, Fritsch EF, Maniatis T (1989) Molecular Cloning: A Laboratory Manual. Cold Spring Harbor, New York: Cold Spring Harbor Laboratory Press.

3. Hanahan D (1983) Studies on transformation of *Escherichia coli* with plasmids. J Mol Biol 166: 557-580.

4. de Lorenzo V, Herrero M, Jakubzik U, Timmis KN (1990) Mini-Tn5 transposon derivatives for insertion mutagenesis, promoter probing, and chromosomal insertion of cloned DNA in Gram-negative eubacteria. J Bacteriol 172: 6568-6572.

5. Blomfield IC, Vaughn V, Rest RF, Eisenstein BI (1991) Allelic exchange in *Escherichia coli* using the *Bacillus subtilis sacB* gene and a temperature-sensitive pSC101 replicon. Mol Microbiol 5: 1447-1457.

6. Cai Z, Lai B, Yun W, Ilinski P, Legnini D, et al. A hard X-ray scanning microprobe for fluorescence imaging and microdiffraction at the advanced photon source. In: Meyer-Ilse W, Warwick T, Attwood D, editors; 2000. AIP. pp. 472-477.
