# Supplementary material for: c-Type Cytochrome-Dependent Formation of U(IV) Nanoparticles by Shewanella oneidensis
Source: PLoS Biol. 2006 Aug 8;4(8):e268. doi: 10.1371/journal.pbio.0040268 (PMC1526764; doi:10.1371/journal.pbio.0040268)
Supplement: Supplementary file 2 [file pbio.0040268.st001.doc]

**Table S1.**

**Bacterial strains and plasmids used for this study**

Strains and plasmids	Description	Reference

*Shewanella oneidensis*

MR-1	Manganese-reducing strain (Lake Oneida, NY)	[1]

MtrC^-^	∆SO1778 (*mtrC*-) deletion derivative of MR-1	This work

OmcA^-^	∆SO1779 (*omcA*-) deletion derivative of MR-1	This work

MtrF^-^	∆SO1780 (*mtrF*-) deletion derivative of MR-1	This work

MtrC^-^/OmcA^-^	∆SO1779 (*omcA*-) deletion within MR-1(*mtrC-*)	This work

CcmC^-^	pMiniHimar RB1 transposon insertion in SO0261	[2]

*(ccmC)*. Requires kanamycin (50 ug/ml) selection.

Also referred to as BG148.

*Escherichia coli*

EC100D pir-116	Host used for construction of pDS3.1	Epicentre

clones. F- *mcrA* Δ(*mrr*-*hsdRMS*^-^ *mcrBC*) φ80d*lacZ*ΔM15 Δ*lacX*74 *recA*1 *endA*1 *araD*139 Δ(*ara*, *leu*)7697 *galU galK* l- *rpsL* *nupG* pir-116

B2155 λpir	Host used for mating with MR-1.	[3]

*thrB*1004 *pro thi strA hsdS lacZ*ΔM15 (F9 *lacZ*ΔM15 *lacl*^q^ *traD*36 *proA*1 *proB*1) Δ*dapA*::*erm* (Erm^r^) *pir*::RP4 (::*kan* (Km^r^) from SM10)

Plasmids

pDS3.1	Suicide vector, Amp^r^, Gm^r^, *sacB*^+^	[4]

pDS3.1::*mtrC*-	pDS3.1 containing sequences that flank *mtrC*	This work

pDS3.1::*omcA*-	pDS3.1 containing sequences that flank *omcA*	This work

pDS3.1::*mtrF*-	pDS3.1 containing sequences that flank *mtrF*	This work

**References**

1. Myers CR, Nealson KH (1988) Bacterial manganese reduction and growth with manganese oxide as the sole electron acceptor. Science 240: 1319–1321.

2. Bouhenni R, Gehrke A, Saffarini D (2005) Identification of genes involved in cytochrome *c* biogenesis in *Shewanella oneidensis*, using a modified mariner transposon. Appl Environ Microbiol 71: 4935-4937.

3. Dehio C, Meyer M (1997) Maintenance of broad-host-range incompatibility group P and group Q plasmids and transposition of Tn5 in *Bartonella henselae* following conjugal plasmid transfer from *Escherichia coli*. J Bacteriol 179: 538-540.

4. Wan XF, Verberkmoes NC, McCue LA, Stanek D, Connelly H, et al. (2004) Transcriptomic and proteomic characterization of the Fur modulon in the metal-reducing bacterium *Shewanella oneidensis*. J Bacteriol 186: 8385-8400.
