# Supplementary material for: c-Type Cytochrome-Dependent Formation of U(IV) Nanoparticles by Shewanella oneidensis
Source: PLoS Biol. 2006 Aug 8;4(8):e268. doi: 10.1371/journal.pbio.0040268 (PMC1526764; doi:10.1371/journal.pbio.0040268)
Supplement: Supplementary file 3 [file pbio.0040268.st002.doc]

**Table S2.**

**Primers used to create the in-frame mutants in this study**

Primer name	Nucleotide sequence*^a^*

∆SO1778 (*mtrC*-)

1778.2_F-O	5’-AAACTGACAACACTTACCCA-3’

1778.2_5-O	5’-TTGCGTGGAAAGCCCACGAA-3’

1778.2_5-I	5’-*TGCCGTGTTGGTGCTTCACA*TGCTGCGAGCAGCAGTGCGA-3’

1778.2_3-I	5’-*TGTGAATCACCAACACGGCA*CCATACCCCAACAGTTGCA-3’

1778.2_3-O	5’-CCATTTGAGTGGGTGACTTGA-3’

1778.2_R-O	5’-GGATTGTGACAGTCGCTA-3’

∆SO1779 (*omcA*-)

1779.2_F-O	5’-GCAACCAACCCATCGATGAC-3’

1779.2_5-O	5’-GCTCCATAGCAGCCAATTTGC-3’

1779.2_5-I	5’-*GACTGGCTTAGGTCGTCTCT*ACCGTTTCATCATGATATTTCC-3’

1779.2_3-I	5’-*AGAGACGACCTAAGCCAGTC*GAAGCACACGGTAACTAAGTCG-3’

1779.2_3-O	5’-GCCTAAGCCTTGCCAGTTAGC-3’

1779.2_R-O	5’-CGTCGAATTTAAAGGTATAGCTA-3’

∆SO1780 (*mtrF*-)

1780_F-O	5’-GAGTAATCCTTGGCTACGTA-3’

1780_5-O	5’-GTGCGAGTTTACATAACGTCGA-3’

1780_5-I	5’-*GGCACCTGTTCATCGTCGTT*TCTATCCTTGGAATACCCTGTC-3’

1780_3-I	5’-*AACGACGATGAACAGGTGCC*CCGACGTACTCAAAGTCCATC-3’

1780_3-O	5’-GAGGTTATCAACGCCTGATGGA-3’

1780_R-O	5’-GTATGTATAACTGTAGCTACCATC-3’

*^a^*Sequences in italics are added to facilitate fusion of first round PCR amplicons as described by Link [1].

**Reference**

1. Link AJ, Phillips D, Church GM (1997) Methods for generating precise deletions and insertions in the genome of wild-type *Escherichia coli*: Application to open reading frame characterization. J Bacteriol 179: 6228-6237.
