# Supplementary material for: c-Type Cytochrome-Dependent Formation of U(IV) Nanoparticles by Shewanella oneidensis
Source: PLoS Biol. 2006 Aug 8;4(8):e268. doi: 10.1371/journal.pbio.0040268 (PMC1526764; doi:10.1371/journal.pbio.0040268)
Supplement: Supplementary file 4 [file pbio.0040268.st003.doc]

**Table S3.**

**Peptide sequences used to produce specific antisera**

Protein (amino acid #s)	Sequence (N → C)

SO1779

OmcA (253-268)	GCHTATSGDPESGNSIE

OmcA (349-364)	GCHTEKPSAHHSSTD

OmcA (684-698)	GCHQKYLSDAAKSHIE

SO1778

MtrC (108-120)	GCLGSSKSYVDNKNG

MtrC (209-222)	CHTQEFADGRGKPH

MtrC (291-302)	GCFAAGKGHSQQLD

MtrC (399-410)	GCKNGALDPKATIN

MtrC (499-514)	GCHTAEFEIHKGKQHAG

MtrC (587-604)	GCSFKKKGALNTAAAADKTG

SO1776

MtrB (23-42)	DGYGLANANTEKVKLSAWSC

MtrB (76-89)	GCNEVAGKFDADLNF

MtrB (90-103)	GCKGEKGYRASVDAYQ

MtrB (201-215)	GCNYMREEKTGLKQASG

MtrB (421-438)	GCYDFKRDQRDYQDRETTDE

MtrB (649-666)	GCRFDYKIENYKDNDAANDI
